# MDMA-assisted therapy is associated with a reduction in chronic pain among people with post-traumatic stress disorder

**DOI:** 10.3389/fpsyt.2022.939302

**Published:** 2022-11-03

**Authors:** Devon Christie, Berra Yazar-Klosinski, Ekaterina Nosova, Pam Kryskow, Will Siu, Danielle Lessor, Elena Argento

**Affiliations:** ^1^Department of Medicine, University of British Columbia, Vancouver, BC, Canada; ^2^MAPS Public Benefit Corporation, San Jose, CA, United States; ^3^British Columbia Centre on Substance Use, Vancouver, BC, Canada; ^4^Department of Health Sciences, Vancouver Island University, Nanaimo, BC, Canada; ^5^MD Inc., Los Angeles, CA, United States; ^6^Memorial University of Newfoundland, St. John’s, NL, Canada

**Keywords:** MDMA-assisted therapy, chronic pain, MDMA, post-traumatic stress disorder, mental health

## Abstract

**Introduction:**

Increasing evidence demonstrates 3,4-methylenedioxymethamphetamine (MDMA)-assisted therapy (MDMA-AT) may be a safe and effective treatment for post-traumatic stress disorder (PTSD). There is growing interest in MDMA-AT to address a range of other health challenges. Chronic pain and PTSD are frequently comorbid, reciprocally interdependent conditions, though the possible role of MDMA-AT in treating chronic pain remains under-investigated. The present analysis examined the impact of manualized MDMA-AT on chronic pain severity among participants with PTSD who were enrolled in a Phase 2 clinical trial investigating MDMA-AT for PTSD (NCT03282123).

**Materials and methods:**

Exploratory data from a subset of participants who completed chronic pain measures (*n* = 32) were drawn from a Phase 2 open-label study sponsored by the Multidisciplinary Association for Psychedelic Studies (MAPS). Multivariable analysis of variance (ANOVA) was utilized to compare pre- vs. post-treatment Chronic Pain Grade Scale (CPGS) values, adjusting for demographics (age, sex, and ethnicity). K-means clustering was then used to group the sample into three clusters to denote high (*n* = 9), medium (*n* = 11), and low (*n* = 12) baseline pain severity, and the same analysis was repeated for each cluster.

**Results:**

Among the 32 participants included in this analysis, 59% (*n* = 19) were women, 72% (*n* = 23) were white, and median age was 38 years [interquartile range (IQR) = 31–47]. Overall, 84% (*n* = 27) reported having pain, and 75% (*n* = 24) reported disability associated with their pain. Significant reductions in CPGS subscales for *pain intensity* and *disability score*, and overall CPGS *severity grade* were observed among participants in the highest pain cluster (*n* = 9, *p* < 0.05), and for *pain intensity* in the medium pain cluster (*n* = 11, *p* < 0.05) post- vs. pre-treatment.

**Discussion:**

Findings demonstrate a high prevalence of chronic pain in this sample of people with severe PTSD and that chronic pain scores among medium and high pain subgroups were significantly lower following MDMA-AT. While these data are preliminary, when considered alongside the frequency of comorbid chronic pain and PTSD and promising efficacy of MDMA-AT for treating PTSD, these findings encourage further research exploring the role of MDMA-AT for chronic pain.

## Introduction

Increasing evidence demonstrates that MDMA-assisted therapy (MDMA-AT) may be a safe and effective treatment for post-traumatic stress disorder (PTSD) (1–4). To date, 11 blinded randomized, controlled Phase 2 and Phase 3 studies of MDMA-AT for PTSD have been published (1–5), and there is growing interest in MDMA-AT for addressing a range of physical and mental health challenges. For instance, pilot studies have explored the potential for MDMA-AT in end of life anxiety (6), social anxiety in autistic adults (7), and alcohol use disorder (8), and exploratory analysis has generated preliminary data for eating disorders (9, 10). However, the possible effect of MDMA-AT on chronic pain remains under-investigated.

Chronic pain is a common, complex disease affecting approximately one-fifth of Americans (11) and up to 40% of people worldwide according to some studies (12) with far-ranging impacts on individuals and society (13). Chronic pain, defined as consistent pain for at least 3 months (14), is a leading cause of disability and is associated with a reduced life expectancy (12). The International Association for the Study of Pain defines pain as “an unpleasant sensory and emotional experience associated with, or resembling that associated with, actual or potential tissue damage” and clearly denotes that the experience of pain is personal and influenced by biological, psychological, and social factors (15). Whereas opioid medications may be utilized to manage acute and/or severe pain such as postoperative and terminal cancer pain, they are largely inappropriate and ineffective to alleviate chronic non-cancer pain and moreover their use is associated with the development of problematic opioid use disorder (OUD) (16). Innovative approaches to managing chronic pain that reduce long term reliance on pharmaceutical interventions are needed. Current best practice guidelines for chronic pain management recommend multidisciplinary treatments that address biological, psychological and social factors that collectively influence a person’s experience of pain (17).

MDMA-AT is a novel intervention under investigation for PTSD that has been granted Breakthrough Therapy status for this indication by the United States’ Food and Drug Administration in 2017 (2). PTSD is a complex, difficult to treat, stress-related psychiatric condition with somatization symptoms that has a profound impact on individuals and society and for which novel treatments are needed (1, 18). In well-established protocols, MDMA-AT combines a program of manualized psychotherapy with administration of MDMA on up to three occasions across the course of treatment in a clinically monitored setting for PTSD (1, 5). Long term analysis of pooled data from recent phase 2 studies of MDMA-AT for chronic, treatment-resistant PTSD demonstrated resolution of PTSD diagnosis in 67% of participants at least 12 months later (3), and phase 3 data demonstrated clinically and statistically significant improvement in severe, chronic PTSD in the MDMA-AT group vs. placebo with therapy combined with an acceptable safety profile (5). MDMA-AT has not yet been investigated to treat chronic pain as an indication.

Chronic pain and PTSD are both challenging to treat, and each contributes to significant dysfunction across all aspects of one’s life despite current available treatments (19, 20). Chronic pain and PTSD are also frequently comorbid; two systematic reviews found prevalence rates of PTSD in clinical pain populations to range from 11.7 to 19.1% (21, 22), and in people with PTSD, prevalence estimates for chronic pain have been as high as 80%, as seen among military personnel (23–25). Both conditions are independently associated with OUD, yet when comorbid are associated with even higher odds of OUD than either condition alone (26). Those with comorbid pain and PTSD also experience greater pain, PTSD symptoms, depression, anxiety, and disability than those with only one of these conditions (27, 28). Chronic pain and PTSD have been described as reciprocally interdependent conditions, with similar maintenance mechanisms such as fear, avoidance, and catastrophizing (29, 30). One literature review notes, “the tight interdependence of symptoms that can be observed in both PTSD and chronic pain syndromes lends support to the idea that these disorders should be situated on the same level, that of a reactive disorder” (31). It has even been suggested that chronic pain might indeed be interpreted as a version of PTSD (32). The Mutual Maintenance Model suggests PTSD and chronic pain exacerbate one another through biases of attention and cognition that create heightened expectations, overestimations, and selective and negative interpretations of both pain-evoking and fear-evoking stimuli. Pain may also serve as a traumatic cue, leading to intrusive PTSD symptoms, and vice versa (33). The Shared Vulnerability Model posits that anxiety sensitivity and genetic predispositions related to the stress response increase the likelihood for certain individuals to develop both conditions (24).

Given the relationship and similarities between chronic pain and PTSD, and anticipated efficacy of MDMA-AT in treating PTSD, MDMA-AT may have potential as a treatment for chronic pain. This notion is further supported by evidence suggesting efficacy for psychotherapeutic approaches in chronic pain (34) and the established importance of biopsychosocial interventions for chronic pain, with MDMA-AT being at once a biological *and* psychological treatment, combining the pharmacological effects of MDMA with psychotherapy. However, there remains a lack of research and data on the effect of MDMA-AT on chronic pain conditions. The present study therefore aimed to explore the potential relationship between MDMA-AT and pain outcomes, drawing on exploratory pain data from a Phase 2 trial of MDMA-AT for severe PTSD (35).

## Materials and methods

Data (December 2017 to August 2019) were drawn from a subset of participants enrolled in a multi-site Phase 2 open-label study known as MP16 (*n* = 33) conducted by MAPS Public Benefit Corporation (MAPS PBC) (35). This study served as a lead-in to Phase 3 studies investigating manualized MDMA-assisted therapy (MDMA-AT) to treat severe PTSD. The researchers designed the study to model the structure of planned Phase 3 trials, and to prepare and supervise sites planning to be part of the Phase 3 studies. The study took place across 12 sites in the United States and followed well-established protocols for MDMA-AT. Details and primary outcomes of the study have been previously described (1).

The primary outcome measure for the MP16 study was the Clinician Administered PTSD Scale according to DSM-5 (CAPS-5), a semi-structured interview addressing PTSD symptom clusters (36). Exploratory data were collected including participants’ Adverse Childhood Experiences (ACE) score at Baseline, and the Chronic Pain Grade Scale (CPGS) questionnaire at Baseline and Study Termination, which were of interest for the present analysis. Eligibility required confirmation of severe PTSD, defined as a CAPS-5 total severity score of 35 or greater. Participants could not have a diagnosis of substance use disorder within 60 days of screening, and psychiatric medications were tapered and discontinued prior to commencing study drug sessions. Participants were allowed to continue concomitant analgesic medications including non-steroidal anti-inflammatory drugs (NSAIDS), acetaminophen, gabapentin, and opiates limited to hydrocodone, morphine and codeine. Participants taking other opiates at enrollment were cross-tapered to an allowable opiate during the preparatory period. Concomitant use of cannabis was prohibited from enrollment to study termination. The protocol for MDMA-AT involved a series of therapy sessions delivered by two clinicians in a “co-therapy” dyad, and participant eligibility and screening were managed by a physician. MDMA administration occurred on three occasions and each session was 8-h in duration in the presence of the co-therapy dyad. These study drug sessions were spaced 3–5 weeks apart and were preceded and/or followed by three non-drug 90-min sessions that served to prepare for and/or facilitate psychotherapeutic integration of each study drug session. According to the flexible dosing schedule, in the first study drug session, all participants received an initial dose of 80 mg MDMA HCl, followed 1.5–2.5 h later by a supplemental dose of 40 mg MDMA HCl. In the second and/or third study drug sessions, participants received an increased dose of 120 mg MDMA HCl followed by a supplemental dose of 60 mg MDMA HCl, 1.5–2.5 h later. All participants followed this dose escalation. The supplemental dose was withheld by the investigator in two instances due to tachycardia and elevated blood pressure following the initial dose, though neither required further medical intervention (35).

The primary outcome for the present analysis was chronic pain, as measured by the CPGS questionnaire, which was administered twice, at Preparatory Session 3 (prior to the first MDMA session), and at study termination which occurred 10–14 weeks after the final MDMA session. Participants were asked to base their responses at study termination on the period since the end of treatment. Secondarily, we looked for associations between chronic pain, childhood adversity and PTSD severity at baseline.

The CPGS questionnaire is a 7-item self-report instrument that measures two dimensions of overall pain severity: pain intensity and pain-related disability, and is valid and reliable for measuring change in severity of chronic pain over time (37). Data encompass current pain, as well as past 6 months pain and pain-related disability and is suitable for use in all chronic pain conditions. *Pain intensity* and *disability score* subscales are combined with *disability points score* to assign a chronic pain *severity grade* (0-IV); individual subscales as well as overall *severity grade* have demonstrated validity and reliability (38) (for definitions of CPGS *severity grade* (0–IV), see Table 1). Demographic variables included age, sex, and ethnicity (white vs. other).

**TABLE 1:** Pain *severity grade* according to CPG.

CPG chronic pain severity grade	Classification method	Definition
0	No pain in prior 6 months	No disability, no pain
1 (I)	Pain Intensity < 50, < 3 Disability Points	Low disability, low intensity
2 (II)	Pain Intensity > 50, < 3 Disability Points	Low disability, high intensity
3 (III)	3–4 Disability Points (regardless of Pain Intensity)	High disability, moderately limiting
4 (IV)	5–6 Disability Points (regardless of Pain Intensity)	High disability, severely limiting

*Severity grade* is classified using the two subscales included in the present analysis (*Pain intensity* 0–100, *disability score* 0–100) plus a *disability points* value (number of disability days in the past 6 months converted to a 0–3-point scale plus *disability score* 0–100 converted to a 0–3-point scale).

### Statistical analysis

Statistical analyses were performed using R version 4.0.3 (R Foundation for Statistical Computing, Vienna, Austria). Bivariate linear and ordinal regression were used to estimate pre-treatment associations between CPGS values, and ACE score, CAPS-5 total severity, and demographic variables.

K-means clustering was used to cluster baseline pain into three groups to denote highest (cluster 1, *n* = 9), medium (cluster 2, *n* = 11), and lowest pain (cluster 3, *n* = 12) within the sample as measured using the CPGS subscale values of *pain intensity*, *disability score*, and the overall pain *severity grade* (39).

To explore the association between MDMA-AT and chronic pain, we compared pre- vs. post-treatment CPGS *pain intensity, disability score*, and composite *severity grade* for all participants (*n* = 32) using *t*-testing for bivariate association and two-way ANOVA for multivariable analysis, adjusting for demographics (sex, age, and ethnicity: white vs. non-white). We hypothesized that only participants reporting medium or high within-sample chronic pain at baseline would experience significant improvement post-treatment, therefore we repeated the same analysis for each CPGS cluster.

## Results

Of the 33 participants enrolled in study MP16, 32 had pre/post CPGS data and were thus included in this analysis. Overall, 59% (*n* = 19) were female, 72% (*n* = 23) were white, and the median age was 38 [interquartile range (IQR) = 31–47] (Table 2). Of this sample, 84% (*n* = 27) reported having pain, and 75% (*n* = 24) reported disability associated with their pain. Participants median ACE score was 4 (IQR = 3–7). No strong associations were found between pre-treatment CPGS values and ACE score, CAPS-5 total severity, or demographics (Table 3).

**TABLE 2:** Baseline demographic variables among participants receiving MDMA-assisted therapy for PTSD included in this analysis (*n* = 32).

Variable	Value	*N* (%)
Sex assigned at birth	Female	19(59.38)
	Male	13(40.63)
Ethnicity	Indigenous (North America)	1 (3.13)
	Asian	5 (15.63)
	Asian and white	1 (3.13)
	Black or African American	1 (3.13)
	Black or African American and white	1 (3.13)
	White	23 (71.88)
Age (median, IQR)		37.5(30.5−46.5)
ACE score (median, IQR)		4(3−7)

**TABLE 3:** Bivariate linear and ordinal regression reveal no strong associations between pre-treatment CPG subscale values and pre-treatment CAPS-5 total severity, ACE score, or demographic variables.

Pain intensity	Bivariate linear regression	*P*-value	Adjusted R squared
	Estimate (95% CI)		
CAPS-5 total severity	0.803 (–0.59, 2.195)	0.249	0.01
ACE score	3.019 (–0.961, 6.999)	0.132	0.04
Sex–male	1.105 (–18.751, 20.961)	0.91	–0.03
Age	–0.654 (–1.518, 0.21)	0.132	0.04
Ethnicity – white	–17.68 (–38.349, 2.988)	0.091	0.06
**Disability score**			
CAPS-5 total severity	–0.078 (–1.747, 1.59)	0.924	–0.03
ACE score	1.438 (–3.378, 6.253)	0.547	–0.02
Sex–male	–2.873 (–26.114, 20.367)	0.802	–0.03
Age	–0.241 (–1.288, 0.807)	0.642	–0.03
Ethnicity – white	–17.249 (–41.835, 7.338)	0.162	0.03

**Severity grade**	**Bivariate ordinal regression estimate**		**Odds ratio (95% CI)**

CAPS-5 total severity	0.02	0.661	1.02 (0.93-1.12)
ACE score	0.127	0.4	1.14 (0.85-1.54)
Sex–male	0.014	0.983	1.01 (0.28-3.72)
Age	–0.027	0.413	0.97 (0.91-1.04)
Ethnicity – white	–0.092	0.199	0.40 (0.09-1.61)

In bivariate *t*-testing and ANOVA, among those in the highest pain cluster mean CPGS values (each of *pain intensity, disability score, and severity grade*) were significantly reduced post-intervention vs. baseline (*n* = 9, *p* < 0.05) (Tables 4–7). A significant reduction in *pain intensity* was observed in the medium pain cluster using ANOVA (*n* = 11, *p* = 0.020; *p* = 0.095 for *t*-test) and there was a trend to reduction for *disability score* and *severity grade* subscales in both bivariate and multivariable analysis (Tables 4, 8).

**TABLE 4:** Bivariate pre-treatment and post-treatment mean and *p*-values using *t*-testing for CPG *pain intensity*, *disability score* and the *severity grade* according to cluster groups (1-high *n* = 9, 2-medium *n* = 11, 3-low *n* = 12), and for the total sample (*n* = 32).

Cluster	Value	Pre-treatment mean	Post-treatment mean	*P*-value
1-High (*n* = 9)	Intensity (0–100)	67	51.9	0.034
	Disability (0–100)	73	38.9	0.004
	Severity grade (0–5)	3.2	2.1	0.019
2-Medium (*n* = 11)	Intensity (0–100)	44.3	31	0.095
	Disability (0–100)	18.7	13.3	0.391
	Severity grade (0–5)	1.3	1.1	0.408
3-Low (*n* = 12)	Intensity (0–100)	9.2	7.2	0.618
	Disability (0–100)	1.4	0.8	0.534
	Severity grade (0–5)	0.6	0.4	0.436
Total sample (*n* = 32)	Intensity (0–100)	37.3	27.8	0.146
	Disability (0–100)	27.7	15.9	0.096
	Severity grade (0–5)	1.6	1.1	0.0117

**TABLE 5:** Multivariable ANOVA, adjusted for demographics (sex male vs. female, age, ethnicity white vs. non-white) for the total sample (*n* = 32).

	Coeff	Sum Sq	*F*-value	*P*-value
**Total sample (*n* = 32)**				
**Intensity**				
Post vs. pre	–9.463	1, 387.877	2.333	0.132
Sex male	–1.594	10.997	0.018	0.892
Age	–0.149	1, 567.046	2.635	0.110
Ethnicity white	–17.074	2, 894.353	4.866	0.031
**Disability**				
Post vs. pre	–11.828	2, 168.380	2.898	0.094
Sex male	–4.445	20.358	0.027	0.870
Age	0.311	2.969	0.004	0.950
Ethnicity white	–16.046	2, 556.382	3.417	0.070
**Severity grade**				
Post vs. pre	–0.452	3.161	2.600	0.112
Sex male	–0.400	1.183	0.973	0.328
Age	0.006	0.239	0.196	0.659
Ethnicity white	–0.658	4.301	3.537	0.065

**TABLE 6:** Multivariable ANOVA, adjusted for demographics (sex male vs. female, age, ethnicity white vs. non-white) for cluster 3-low (*n* = 12).

	Coeff	Sum Sq	*F-*value	*P*-value
**Cluster 3–low (*n* = 12)**				
**Intensity**				
Post vs. pre	–1.945	22.698	0.254	0.620
Sex male	0.895	4.779	0.053	0.820
Age	–0.193	186.798	2.087	0.165
Ethnicity white	–4.104	62.434	0.697	0.414
**Disability**				
Post vs. pre	–0.555	1.848	0.454	0.509
Sex male	0.651	0.846	0.208	0.654
Age	–0.011	6.078	1.492	0.237
Ethnicity white	–2.174	17.526	4.301	0.052
**Severity grade**				
Post vs. pre	–0.167	0.167	0.639	0.434
Sex male	–0.054	0.171	0.657	0.428
Age	–0.015	0.693	2.655	0.120
Ethnicity white	–0.052	0.010	0.039	0.846

**TABLE 7:** Multivariable ANOVA, adjusted for demographics (sex male vs. female, age, Ethnicity white vs. non-white) for cluster 1-high (*n* = 9).

	Coeff	Sum Sq	*F*-value	*P*-value
**Cluster 1-high (*n* = 9)**				
**Intensity**				
Post vs. pre	–15.187	1, 037.857	7.279	0.018
Sex male	–7.589	100.067	0.702	0.417
Age	–0.755	1, 123.557	7.880	0.015
Ethnicity white	0.919	1.972	0.014	0.908
**Disability**				
Post vs. pre	–34.073	5, 224.464	10.396	0.007
Sex male	–4.300	123.432	0.246	0.628
Age	0.120	74.018	0.147	0.707
Ethnicity white	2.383	13.259	0.026	0.873
**Severity grade**				
Post vs. pre	–1.111	5.556	8.867	0.011
Sex male	–0.910	4.000	6.385	0.025
Age	–0.006	0.023	0.037	0.850
Ethnicity white	0.344	0.277	0.441	0.518

**TABLE 8:** Multivariable ANOVA, adjusted for demographics (sex male vs. female, age, ethnicity white vs. non-white) for the cluster 2-medium (*n* = 11).

	Coeff	Sum Sq	*F-*value	*P*-value
**Cluster 2-medium (*n* = 11)**				
**Intensity**				
Post vs. pre	–13.332	888.711	6.810	0.020
Sex male	–0.345	979.720	7.507	0.015
Age	0.762	126.519	0.969	0.340
Ethnicity white	–31.285	1, 938.220	14.851	0.002
**Disability**				
Post vs. pre	–5.334	142.258	0.953	0.344
Sex male	7.906	802.138	5.375	0.035
Age	0.273	21.379	0.143	0.710
Ethnicity white	–10.265	208.663	1.398	0.255
**Severity grade**				
Post vs. pre	–0.200	0.200	2.763	0.117
Sex male	0.144	1.800	24.863	0.000
Age	0.018	0.005	0.063	0.805
Ethnicity white	–1.032	2.109	29.138	0.000

## Discussion

The present study found a high prevalence of pain among this sample of individuals with PTSD, in keeping with other published data demonstrating high rates of comorbid pain and PTSD (40). Statistically significant within subjects reductions in CPGS subscales including *pain intensity, disability score, and* composite chronic pain *severity grade* were observed following MDMA-AT among participants in the highest pain cluster (*n* = 9), and for *pain intensity* only in the medium pain cluster (*n* = 11), suggesting that MDMA-AT may be associated with reduction in chronic pain and pain-related disability. Significant reductions in pain and pain-related disability among participants in the highest pain cluster may be due to higher baseline pain values allowing greater margin for improvement; there may also be a mechanism related to an amygdala-based threat response to higher levels of pain being positively impacted by MDMA-AT, since overlapping brain areas have been shown to be active in both pain related threat perception, and anxiety and fear-based threat perception as typified by PTSD (32). Interestingly, among this sample baseline pain severity and PTSD severity were not positively correlated (Table 3). We speculate that differences in pain perception among individuals with PTSD symptoms may account for this finding; for example, high PTSD severity may distract from pain symptomatology, and vice versa, but this warrants further investigation. Previous research has demonstrated a unique and paradoxical pain perception pattern (hypo-responsiveness and hyper-sensitivity) in experimental and chronic pain among people with PTSD: PTSD-related dissociation was associated with higher pain threshold, whereas supra-threshold pain stimulus ratings were higher in those with PTSD in comparison to controls. Pain hyper-responsiveness was positively associated with anxiety sensitivity, but negatively correlated with dissociation levels (41). Anxiety and dissociation are intercorrelated in PTSD (41) yet their relationship to pain perception among people with PTSD has not been systematically studied and warrants further investigation. To our knowledge, this is the first study to demonstrate an association between MDMA-AT and reduced pain among individuals with PTSD.

Chronic pain is an international public health emergency for which new treatments are desperately needed (42, 43). While chronic pain is frequently comorbid with PTSD, few studies have examined treatments designed to effectively address both conditions concurrently (40), yet when outcomes are assessed related to psychological interventions for both disorders in individual studies, moderate effects for PTSD and small effects for pain emerge, suggesting transdiagnostic effects (44). International best practice recommendations involve understanding and addressing chronic pain within a biopsychosocial model, with personalized multidisciplinary treatment approaches that include both physical and psychological interventions (42, 43). Many chronic pain conditions, such as fibromyalgia, tension headache, and non-specific low back pain, involve alterations in central nervous system pain pathways leading to abnormal processing of pain signals, and are known collectively as “nociplastic” type pain. History of psychological, emotional, sexual, or physical abuse or a combination of these predispose to nociplastic pain (45). These conditions tend to respond poorly to physical treatment (medication or procedural interventions), and more readily to non-pharmacological approaches, such as educational, behavioral and psychotherapy interventions (46).

MDMA is a psychoactive compound that promotes serotonin release, and may exert therapeutic effects by enhancing fear memory extinction (47), promoting greater self-compassion (48), reducing self-criticism (48), reducing PTSD-related shame and anger (49) and causing acute prosocial and interpersonal effects that support the quality of the therapeutic alliance in psychotherapy (50). MDMA promotes oxytocin release, which helps increase interpersonal focus, feelings of interpersonal trust and social affiliation (51–53), and has been shown to reduce pain associated with social rejection (54). MDMA-AT has been associated with changes in personality persisting at two-month follow-up, notably increased personality trait Openness and reduction in Neuroticism (55). It has been suggested that MDMA may serve to acutely widen a “window of tolerance” in autonomic regulatory capacity which, combined with therapy, enables participants to approach and reprocess highly charged traumatic content without becoming overwhelmed or limited by hyperarousal or dissociative processes (5). MDMA may also re-open oxytocin-dependent “critical period” neuroplasticity in social learning that may also function to enhance or accelerate therapeutic change (56).

Given the high comorbidity of PTSD and chronic pain reflected in the present analysis, and the growing literature suggesting shared mechanisms both predisposing to and maintaining both conditions, it is conceivable that some of these proposed mechanisms explaining the efficacy of MDMA-AT for PTSD may be relevant for treating chronic pain. In particular, MDMA-AT effects related to enhanced fear extinction and approaching rather than avoiding negatively charged content may be relevant to fear and avoidance phenomena that are known to intensify and perpetuate chronic pain (32). Self-compassion, enhanced through MDMA, has also been linked with improved pain outcomes (57). Treatment with an existential focus may play an important role in chronic pain recovery (42, 58) particularly when combined with other psychological and pharmacological approaches (40) and has been shown to be effective in decreasing pain-related disability (59). MDMA-related improvements in intrapersonal attitudes including reduced self-criticism may impart benefit to chronic pain-associated existential challenges to related to identity, social roles, meaning and purpose, which are relevant yet underrepresented areas of treatment focus for chronic pain (40). Interpersonal effects that enhance therapeutic alliance may also be of relevance since psychological interventions for chronic pain require a strong therapeutic relationship to maximize benefit (12). Additionally, MDMA-enhanced perception of social connection and support may target chronic pain-related social isolation, a factor that exacerbates pain symptomatology, and when reduced, is associated with improvements in emotional and physical functioning among chronic pain patients (60). The potential for MDMA-AT to promote change in personality including reduced trait Neuroticism may also be of relevance, since neuroticism predicts poor adjustment to chronic pain (61).

Our data demonstrate a high median ACE score among this sample (4; IQR 3–7) and are consistent with previous research in the intersecting fields of chronic pain and trauma, that indicate an association with adverse childhood experiences (ACE) (13, 62). Stress response psychobiology is of particular relevance to this discussion. While short term stress responses support an individuals’ immediate survival, chronic adversity and/or repeated stressors, particularly during vulnerable developmental periods, can lead to long term changes to the structure and function of hormonal, neurological and psychological systems in ways that predispose to poor health outcomes (63–65), such as chronic pain. The capacity to tolerate increased levels of stress and to recover quickly, known as resilience, relies upon optimal environmental conditions whereby interacting psychobiological systems can develop ideal responses, and healthy attachment relationships are of particular importance (66). The potential of MDMA to re-open critical period neuroplasticity related to social reward learning, combined with interpersonal effects such as enhanced empathy and trust, may create optimal conditions within the MDMA-AT therapeutic relationship to improve resilience and thus create more favorable conditions for recovering from chronic pain and other chronic conditions that are negatively impacted by stress.

### Strengths and limitations

K-means clustering provided a simple and efficient method to define groups according to pre-treatment CPGS values representing high, medium, and low within-sample pain values, which allowed for analysis using regression, *t*-testing and ANOVA for the whole sample and then separately for each of these stable clusters. The small sample size (*n* = 32) limited the power of these analyses, especially for the high pain cluster (*n* = 9) that demonstrated significant post-treatment reductions in pain values. Analysis was not conducted according to a hierarchical testing strategy, introducing the possibility for Type 1 error. Despite these limitations, participants in this sample were not subject to expectation bias regarding any positive impact of MDMA-AT on pain, since participants were recruited to the original study for experimental treatment of PTSD, whereas CPGS was administered and collected as exploratory data only. These data encourage further research with a larger sample in randomized, controlled trials in which the intervention is specifically investigated as a treatment for chronic pain.

In this sample, MDMA was administered 3 times according to a flexible dosing schedule; however, the analysis was not powered to detect differences in total MDMA dose given that the vast majority of participants received the escalation dose (120 mg HCl for the second and/or third dosing session) and supplemental dose of 40 or 60 mg HCl. Future studies could address exposure-response analyses on change in chronic pain.

While the CPGS is a valid and reliable instrument, recall bias may limit accuracy of self-report responses. Further, the CPGS version administered to participants included one question (#5) that differed from the published version of the measure: on the 10-point scale, 10 was defined for this analysis as *10* = *Extreme Change*, rather than *10* = *Unable to Carry on Any Activities* in response to the question *In the past 6 months how much has pain interfered with your daily activities rated on 0-10 scale?* Therefore, while participants were aware the 10-point scale asked for a rating of severity of pain interference with daily activities, they might have interpreted *extreme change* as still allowing for minimal activity, rather than absolute inability to carry on any activities. This could theoretically lead to false elevation of baseline and post-intervention *disability score*, which could likewise falsely elevate total *severity score*.

Associations between MDMA-AT and chronic pain may be influenced by confounding variables not examined in the present study, such as concurrent analgesic medications. Furthermore, participants in study MP16 were not selected for experiencing chronic pain, rather they were selected for having PTSD symptoms for at least 6 months, therefore some participants may have completed the questionnaire in reference to pain experienced for a shorter period. This limits generalizability of these exploratory data to true chronic pain conditions that are not comorbid with PTSD. In addition, different etiologies for pain could lead to differing pain or disability perceptions, or different secondary responses to the MDMA-AT for PTSD intervention. Within study population and within cluster distribution of pain by etiology could lead to further information and enhance future research protocols investigating MDMA-AT for chronic pain that include a mixed sample.

This study included predominantly white participants, which limits the generalizability of the findings to other populations. Not all people are affected by chronic pain equally; most data indicate higher prevalence in racialized and marginalized populations including African American and Indigenous people, women, and individuals from lower socioeconomic backgrounds (12). Further research should aim to increase diversity among research participants, as well as consider how chronic pain is experienced across the lifespan and across cultures.

## Conclusion

These findings demonstrate a high prevalence of pain in this sample of participants with severe PTSD, and that pain intensity, disability, and a composite pain severity index grade among those with the highest pain were significantly lower following MDMA-AT. While these data are limited and primarily hypothesis generating, they suggest that MDMA-AT may be associated with reductions in pain and pain-related disability among individuals with PTSD. Despite being derived from an exploratory endpoint, these findings when considered alongside the promising efficacy of MDMA-AT for treating PTSD and the transdiagnostic similarities between PTSD and chronic pain, support advancement of research exploring the role of MDMA-AT as a novel intervention for chronic pain and PTSD/chronic pain co-morbidity.

## Data availability statement

The datasets analyzed for this study were accessed by request to the MAPS Public Benefit Corporation (MAPS PBC). Restrictions apply to the availability of these data, which were used under license for the current study, and so are not publicly available. Data are, however, available upon reasonable request and with the permission of MAPS at http://maps.org/datause. All requests for raw and analyzed data are promptly reviewed by MAPS PBC to verify if the request is subject to any confidentiality obligations. Patient-related data not included in this publication were generated as part of clinical trials and may be subject to patient confidentiality. Any data that can be shared may be released via a data use agreement.

## Ethics statement

The MP16 clinical trial was reviewed and approved by the Western Copernicus Group Independent IRB (Research Triangle, NC, United States), Western IRB (Puyallup, WA, United States), University of California San Francisco Human Resource Protection Program IRB, University of Washington Human Subjects Division IRB, and University of British Columbia Providence Health Care Research Ethics Board. Participants provided their written and informed consent for participation in MP16.

## Author contributions

BY-K: substantial contributions to the conception and design of study MP16. DC and EA: data analysis, plan conception, and design. DC: initial draft manuscript preparation. EN, DC, and EA: analysis and interpretation of the results. PK, WS, DL, and BY-K: review of data analysis and interpretation. DC, EA, PK, WS, DL, and BY-K: critical and final review of the manuscript. All authors contributed to the article and approved the submitted version.
